# A CT-based deep learning model to predict local recurrence-free survival in primary retroperitoneal sarcoma

**DOI:** 10.3389/fmed.2025.1725377

**Published:** 2026-01-02

**Authors:** Yaru Ren, Ziyang Xue, Ting Liang, Yanjun Sun, Jianhong Gao, Ke Liu, Junxiang Zhang, Jie Lian, Xuqi Li, Shufeng Wang

**Affiliations:** 1 Department of General Surgery, The First Affiliated Hospital of Xi'an Jiaotong University, Xi An, Shaanxi, China; 2 Department of Surgical Oncology, Xi'an No. 3 Hospital, The Affiliated Hospital of Northwest University, Xi An, Shaanxi, China; 3 Department of Medical Imaging, The First Affiliated Hospital of Xi'an Jiaotong University, Xi An, Shaanxi, China; 4 Department of Pathology, The First Affiliated Hospital of Xi'an Jiaotong University, Xi An, Shaanxi, China

**Keywords:** deep learning, local recurrence, prognosis prediction, radiomics, retroperitoneal sarcoma

## Abstract

**Background:**

Survival prediction using radiomics and deep learning (DL) has shown promise, but its utility for predicting local recurrence among patients with primary retroperitoneal sarcoma (RPS) remains unexplored. This study sought to construct a DL framework leveraging preoperative CT to predict local recurrence-free survival (LRFS) in RPS.

**Methods:**

We retrospectively enrolled 115 primary RPS patients (2013–2024), splitting into training (*N* = 86) and validation (*N* = 29) sets. An end-to-end DL model was designed to forecast LRFS using contrast-enhanced CT images. The DL-based score (DL-score) was contrasted with a conventional handcrafted radiomics model (Rad-score) and clinical model. Integrated models combining DL-score or Rad-score with clinicopathological factors were constructed (DLCM and RSCM). Model evaluation included the C-index, time-dependent ROC, calibration, decision curve analysis and survival analysis.

**Results:**

The DL-score outperformed Rad-score and clinical model, yielding higher C-index (training: 0.778 vs. 0.716 vs. 0.721; validation: 0.730 vs. 0.654 vs. 0.648). The DL-score proved to be an independent predictor of LRFS in training sets (adjusted HR = 5.950, 95% CI: 2.800–12.644; *p* < 0.001) and effectively categorized patients into high- and low-risk categories (*p* < 0.0001, *p* = 0.012, respectively). The combined DLCM further improved the performance, attaining C-index of 0.848 (95% CI: 0.790–0.915) and 0.749 (95% CI: 0.601–0.878) in the training and validation sets, respectively. The DLCM exhibited strong calibration and clinical utility and was an effective prognostic tool for risk classification in both cohorts.

**Conclusions:**

The CT-based DL model effectively predicts LRFS preoperatively in RPS, aiding risk stratification and guiding individualized therapeutic strategies.

## 1 Introduction

Retroperitoneal sarcoma (RPS) is an uncommon mesenchymal malignancy originating from the retroperitoneal compartment, with an extremely low yearly occurrence rate of 2.7 new cases per million population (1). Surgical excision stands as the sole curative treatment for RPS. Even after complete resection, however, the local recurrence rate remains high at 30%−70% (2–5), with up to 75% of deaths taking place without distant metastasis (6).

Early screening of RPS patients with elevated risk of local recurrence is essential for guiding treatment decisions and surveillance protocols. Current prognostic factors mainly rely on clinical indicators, including tumor size, histologic subtype, pathological grade, and resection completeness (2, 5, 7). Nevertheless, due to the marked heterogeneity of RPS, the predictive performance of existing clinical models remains suboptimal.

Computed tomography (CT) is routinely used for imaging evaluation in RPS (8), yet the difficulty in quantifying imaging features has limited their integration into survival prediction models, leaving the abundant tumor characteristics embedded in imaging underutilized. Radiomics offers a non-invasive means to characterize tumor biology by mining high-throughput quantitative information from medical images (9). It could capture lesion and tissue features—such as tumor heterogeneity and morphology—that are often imperceptible by the human eye. With progress in computer vision, deep learning (DL) involving convolutional neural networks (CNNs) (10) enables the automatic extraction of complex and comprehensive signatures directly from raw images and generates task-specific representations, without requiring explicit feature engineering, thereby further enhancing predictive modeling. Both radiomics and DL have shown remarkable efficacy in precision diagnosis, treatment response prediction, and prognostic assessment across multiple cancers (11–15). Recently, several studies (16–18) have illustrated the potential of radiomics and DL in predicting histologic subtype and grade, overall survival (OS), and postoperative distant metastasis in RPS. However, to our knowledge, no studies to date have established an end-to-end DL model utilizing CT images to forecast local recurrence-free survival (LRFS) among RPS patients.

Therefore, this study seeks to establish and verify a DL model using preoperative contrast-enhanced CT to predict LRFS in RPS patients following surgery. Furthermore, we sought to construct an integrated multimodal model by integrating the DL model and clinical factors to further enhance predictive performance.

## 2 Patients and methods

### 2.1 Patients

This retrospective investigation obtained ethics authorization from the Ethics Committee of the First Affiliated Hospital of Xi'an Jiaotong University (XJTU1AF2025LSYY-717), which also waived the requirement for informed consent. Specific inclusion and exclusion criteria are provided in Supplementary Content 1. Finally, 115 individuals were enrolled in this study between March 2013 and March 2024. These participants were randomly split into a training set (*n* = 86) and a validation set (*n* = 29) in a 3:1 ratio. The primary outcome was local recurrence-free survival (LRFS), calculated from surgery until local abdominal recurrence, death, or the last follow-up. The cutoff time for censoring purposes was March 2025.

### 2.2 Clinical model architecture

Clinicopathologic data were retrieved from hospital records, encompassing gender, age, body mass index (BMI), tumor size, multifocality, histologic subtype, Federation Nationale des Centers de Lutte Contre le Cancer (FNCLCC) grade, and adjuvant treatment. Univariate Cox regression was initially conducted to assess each clinical variable. Factors showing significance (*p* < 0.05) in univariate screening were entered into a multivariable Cox analysis. Bidirectional stepwise selection governed by the Akaike Information Criterion (AIC) was utilized for the multivariate regression. Ultimately, only variables demonstrating independent prognostic value were retained to construct the clinical model.

### 2.3 Image collection and preprocessing

Contrast-enhanced CT images in arterial phase were retrieved from the Picture Archiving and Communication System (PACS) in Digital Imaging and Communications in Medicine (DICOM) format. Detailed scanner parameters are included in Supplementary Content 2. All slices underwent resampling to a consistent voxel spacing of 1 × 1 × 5 mm^3^ via B-spline interpolation. To locate tumor areas, a cubic bounding box was positioned to cover the largest axial cross-sectional area of each lesion. Within this volume, regions of interest (ROIs) were manually annotated slice-by-slice along tumor boundaries on arterial phase images (5-mm slice thickness) in a blinded manner by Radiologist 1 (with 5 years of sarcoma experience) utilizing 3D Slicer (version 5.0.3). To evaluate segmentation consistency, 28 randomly selected patients' scans were re-delineated 1 month later by Radiologist 1 and Radiologist 2 (3 years' experience in sarcoma) to compute interclass and intraclass correlation coefficients (ICCs). When discrepancies arose, a senior radiologist (with experience in RPS imaging over 15 years) reassessed to make a final decision.

### 2.4 Handcrafted radiomics model establishment

Handcrafted radiomics features were obtained from the segmented three-dimensional ROIs utilizing the corresponding segmentation masks. Feature extraction was carried out with the Pyradiomics toolkit (v3.0.1; Python 3.12) following z-score normalization, yielding 1,688 features (encompassing shape, first-order, texture, and filter-based types). More descriptions of features extraction are available in Supplementary Content 3. Within the training cohort, ICCs analysis, univariate Cox regression, Spearman correlation testing and least absolute shrinkage and selection operator (LASSO) Cox regression were employed for selecting radiomic features. The radiomics score (Rad-score) was then formulated as a linear integration of the selected features weighted by their multivariate Cox coefficients. Detailed information on the radiomics features selection and Rad-score model construction are given in Supplementary Content 4.

### 2.5 DL model construction

For each patient, a set of five consecutive slices was selected, centered on the layer with the maximal cross-sectional tumor area, with two slices above and two below. Each slice was normalized using an adaptive windowing strategy followed by z-score normalization. All images were resized to 256 × 256 pixels and transformed into single-channel tensors. For enhancing model generalizability and preventing overfitting, extensive data augmentation was performed during training, which encompassed random horizontal and vertical flipping, rotations, affine transformations (translation, scaling, shearing), resizing, normalization and random erasing on tensor images (19).

**Figure 1:**
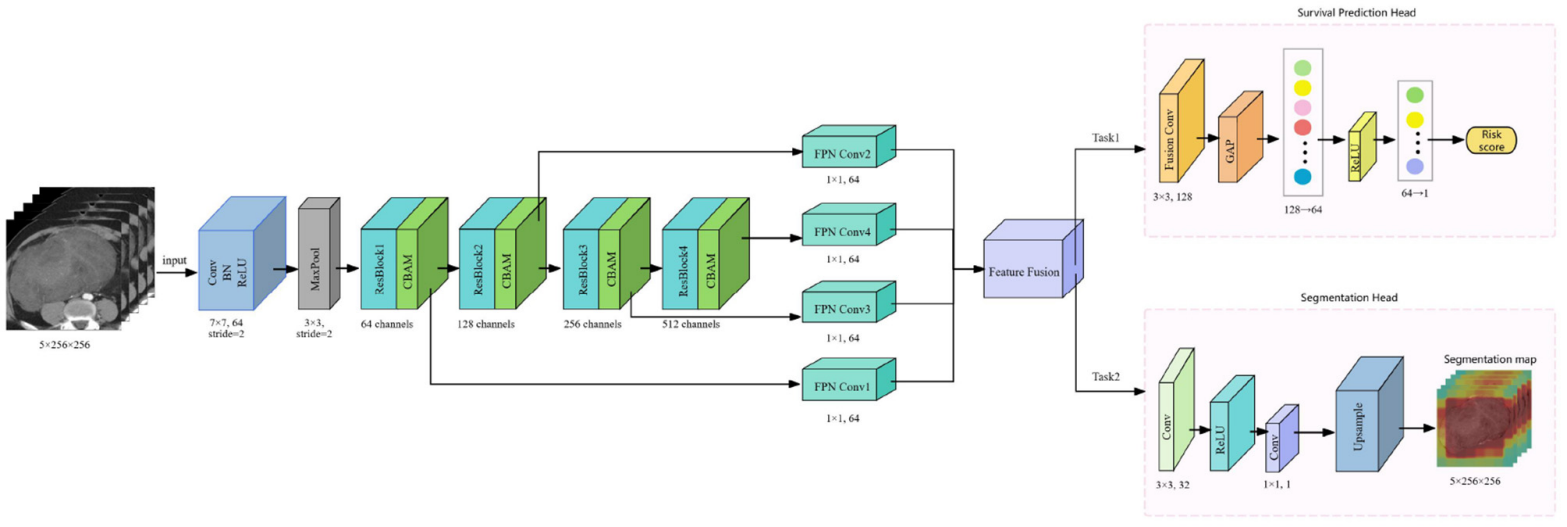
Workflow of the DL model. DL, deep learning; Conv, convolution; BN, batch normalization; ReLU, rectified linear unit; MaxPool, max pooling; ResBlock, residual block; CBAM, convolution block attention module; FPN, feature pyramid network; GAP, global average pooling.

The workflow of the DL model is outlined in Figure 1. We designed an end-to-end CNN for survival analysis based on a modified ResNet-18 (20) backbone pretrained on ImageNet. The inputs consisted of ROIs centered on the tumor using corresponding lesion masks. A convolutional block attention module (CBAM) (21) (Supplementary Content 5) was incorporated after each main layer to enhance feature representation and perceptual ability of the network. A multi-scale feature pyramid was constructed using 1 × 1 convolutions and a multi-scale feature fusion approach, which incorporates high-level semantic data and low-level spatial details (22). The fused features underwent global adaptive average pooling and were subsequently input into fully connected layers to produce a risk score (DL-score) for each patient. Additionally, an auxiliary segmentation head was incorporated to predict tumor masks for each slice, facilitating multi-task learning and thus boosting the model's ability to represent tumor complexity and improving prognostic performance (23). The composite loss function comprised Cox proportional hazards loss (24) (Supplementary Content 6) for survival prediction and a weighted dice loss (25, 26) to enhance tumor segmentation, with a weighted ratio of 1:0.15. The model yielding the highest concordance index (C-index) on the validation set was chosen for final analysis. For interpretability, gradient-weighted class activation maps (Grad-CAM) (27) derived from the last convolutional layer were used to visualize lesion regions contributing most to risk predictions.

Model training was conducted with PyTorch on a NVIDIA GeForce RTX 4060 8GB GPU. The initial learning rate was configured as 2 × 10^−4^ and regulated via cosine annealing with warm restarts. Optimization was carried out with the AdamW optimizer (28) over a maximum of 120 epochs, using a batch size of 16. Mixed precision (AMP) and gradient accumulation were utilized to optimize GPU efficiency and stabilize the training process. Early stopping was triggered based on the validation C-index, with a patience of 30 epochs.

### 2.6 Combined model development

To integrate clinical and imaging-based predictors, a multivariate Cox proportional hazards regression was performed within the training dataset using following variables: sex, age, BMI, tumor size, multifocality, histologic subtype, FNCLCC grade, adjuvant treatment, Rad-score, and DL-score. Variables showing statistical significance in the multivariate analysis were identified as independent prognostic predictors and retained in the combined model. To separately assess the added prognostic value of the Rad-score and DL-score beyond the clinical model, two combined models were developed via Cox regression. The radiomics-clinical combined model (RSCM) was established by incorporating the Rad-score alongside significant clinical predictors. Similarly, the deep learning-clinical combined model (DLCM) was formulated by combining the DL-score with independent clinical factors.

### 2.7 Statistical analysis

The discriminative ability of the proposed models was evaluated via Harrell's C-index and time-dependent area under the curve (AUC) obtained from receiver operating characteristic (ROC) analyses. Calibration plots with 1,000 bootstrap resamples served to visualize the agreement between model-predicted probabilities and observed outcomes. Decision curve analysis (DCA) was applied to estimate the clinical usefulness of each model by computing the net benefit across different probability thresholds. Univariate and multivariate Cox regression analyses were conducted to identify prognostic values of distinct variables. Kaplan–Meier survival analyses were generated for high- and low-risk groups, stratified by the median risk score calculated from the DL and DLCM model within the training cohort (29). Group differences were compared using log-rank tests.

For continuous variables, the Student's *t*-test or Mann–Whitney *U* test was employed; categorical variables were compared using chi-square or Fisher's exact test, and ordinal variables were analyzed with the Kruskal–Wallis test. Data analyses were performed with R Studio (version 4.4.3), SPSS (IBM version 25.0), or Python (version 3.9). A two-sided *p* values < 0.05 was considered statistically significant.

## 3 Results

### 3.1 Patient characteristics

The baseline data for all included patients are summarized in Table 1. 115 individuals were included in our study, comprising 86 eligible patients in the training set [39 male and 47 female; mean age, 54.66 ± 13.05 years; median follow-up time, 24 (14.0–43.8) months] and 29 eligible patients in the validation set [12 male and 17 female; mean age, 52.93 ± 12.46 years; median follow-up time, 21 (12.0–45.0) months]. The clinical characteristics of the two cohorts were comparable, and no notable significant difference was detected.

**Table 1:** Patient characteristics.

**Characteristics**	**Training set**	**Validation set**	***p*-Value**
	***N*** = **86**	***N*** = **29**	
**Gender**			
Male	39 (45.3%)	12 (41.4%)	0.876
Female	47 (54.7%)	17 (58.6%)	
Age (years)	54.66 ± 13.05	52.93 ± 12.46	0.489
BMI	23.15 ± 3.11	22.70 ± 3.07	0.450
Tumor size (cm)	15.30 (8.68–25.87)	14.00 (7.57–22.45)	0.288
**Multifocality**			
No	73 (84.9%)	22 (75.9%)	0.409
Yes	13 (15.1%)	7 (24.1%)	
**Histologic subtype**			
WDLPS	28 (32.6%)	8 (27.6%)	0.872
DDLPS	11 (12.8%)	3 (10.3%)	
LMS	13 (15.1%)	6 (20.7%)	
Others	34 (39.5%)	12 (41.4%)	
**FNCLCC grade**			
G1	23 (61.6%)	16 (55.2%)	0.440
G2	24 (27.9%)	8 (27.6%)	
G3	9 (10.5%)	5 (17.2%)	
**Adjuvant treatment**			
No	64 (74.4%)	19 (65.5%)	0.493
Yes	22 (25.6%)	10 (34.5%)	
**Outcome events**			
No	45 (52.3%)	15 (51.7%)	0.955
Yes	41 (47.7%)	14 (48.3%)	
Median follow-up time (months)	24 (14.0–43.8)	21 (12.0–45.0)	0.562

WDLPS, well-differentiated liposarcoma; DDLPS, dedifferentiated liposarcoma; LMS, leiomyosarcoma; FNCLCC, Federation Nationale des Centers de Lutte Contre le Cancer. Outcome event was defined as abdominal local recurrence or death.

### 3.2 Performance of the clinical model

Supplementary Table S1 displays the findings of multivariate Cox analysis used to identify independent clinical variables. The model was built based on multifocality and FNCLCC grade. Within the training dataset, the C-index of this clinical model achieved 0.721 [95% confidence interval (CI): 0.658–0.800], while in the validation set it was 0.648 (95% CI: 0.513–0.784; Table 2). Furthermore, the clinical model could accurately forecast 1-, 3-, 5-years LRFS, with AUC in the validation set of 0.748 (95% CI: 0.563–0.933), 0.675 (95% CI: 0.454–0.895) and 0.771 (95% CI: 0.575–0.966; Supplementary Figure S1).

**Table 2:** The performance of different models in predicting LRFS.

**Cohorts**	**Models**	**C-index**	**1-years AUC**	**3-years AUC**	**5-years AUC**
Training set	Clinical	0.721 (0.658–0.800)	0.816 (0.715–0.916)	0.793 (0.687–0.900)	0.773 (0.654–0.892)
	Rad-score	0.716 (0.611–0.804)	0.736 (0.599–0.873)	0.742 (0.612–0.872)	0.804 (0.682–0.927)
	DL-score	0.778 (0.708–0.848)	0.761 (0.637–0.885)	0.838 (0.736–0.940)	0.900 (0.812–0.988)
	RSCM	0.792 (0.734–0.868)	0.838 (0.734–0.941)	0.857 (0.767–0.947)	0.912 (0.836–0.987)
	DLCM	0.848 (0.790–0.915)	0.842 (0.741–0.944)	0.933 (0.877–0.989)	0.976 (0.940–1.000)
Validation set	Clinical	0.648 (0.513–0.784)	0.748 (0.563–0.933)	0.675 (0.454–0.895)	0.771 (0.575–0.966)
	Rad-score	0.654 (0.471–0.808)	0.699 (0.485–0.913)	0.589 (0.339–0.838)	0.682 (0.423–0.941)
	DL-score	0.730 (0.594–0.875)	0.865 (0.727–1.000)	0.855 (0.673–1.000)	0.749 (0.519–0.979)
	RSCM	0.662 (0.476–0.812)	0.714 (0.507–0.922)	0.605 (0.355–0.856)	0.702 (0.444–0.961)
	DLCM	0.749 (0.601–0.878)	0.910 (0.798–1.000)	0.860 (0.672–1.000)	0.785 (0.553–1.000)

LRFS, local recurrence-free survival; AUC, area under the receiver operating characteristic curve; Rad-score, radiomics score; DL-score, deep learning score; RSCM, the model combined Rad-score with clinical factors; DLCM, the model combined DL-score with clinical factors.

### 3.3 Performance of the Rad-score model

Two most valuable radiomics features (logarithm_firstorder_Entropy and wavelet.LLH_glszm_GrayLevelNonUniformityNormalized) were chosen for building the handcrafted radiomics model (Supplementary Figure S2). The Rad-score model attained a C-index of 0.716 (95% CI: 0.611–0.804) within the training dataset and 0.654 (95% CI: 0.471–0.808) within the validation dataset (Table 2). In the validation cohort, this Rad-score model accurately predicts LRFS at 1, 3, and 5 years, with AUC of 0.699 (95% CI: 0.485–0.913), 0.589 (95% CI: 0.339–0.838) and 0.682 (95% CI: 0.423–0.941; Supplementary Figure S3).

### 3.4 Performance of the DL model

**Figure 2:**
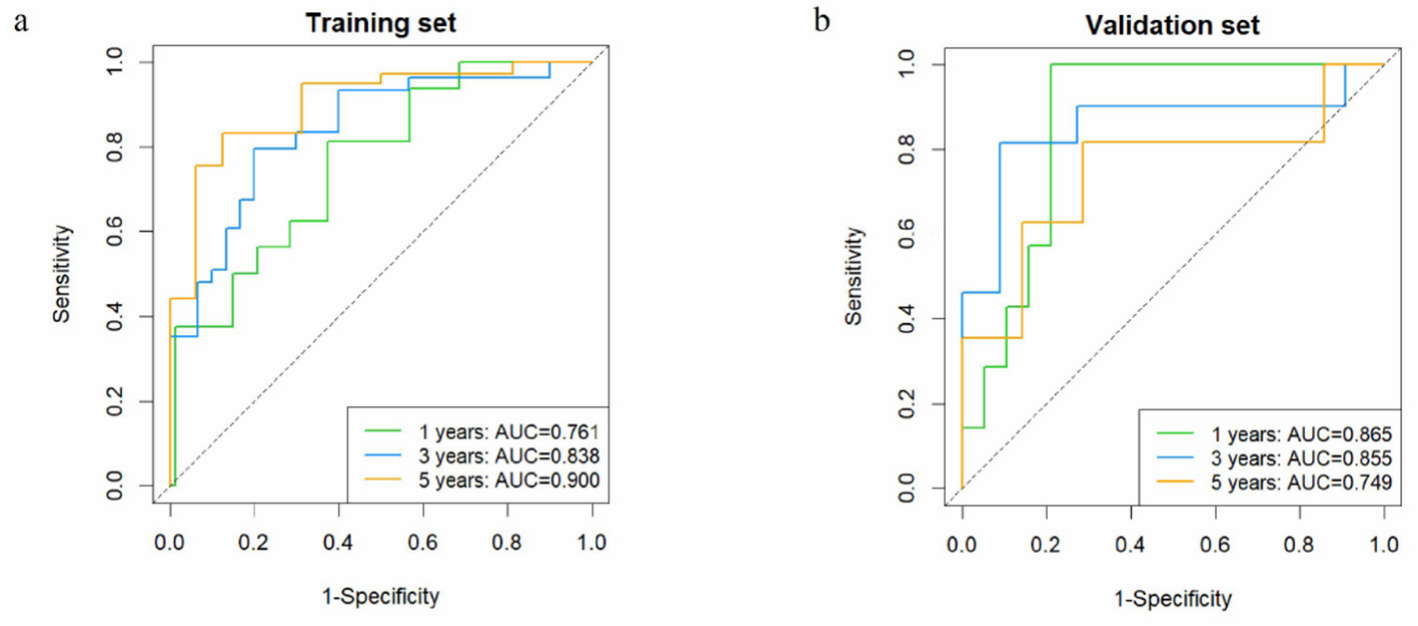
Predictive efficacy of DL model on LRFS. Time-dependent ROC curves of the DL-score in the training **(a)** and the validation **(b)** cohorts. DL, deep learning; LRFS, local recurrence-free survival; ROC, receiver operating characteristic; AUC, area under the curve.

**Figure 3:**
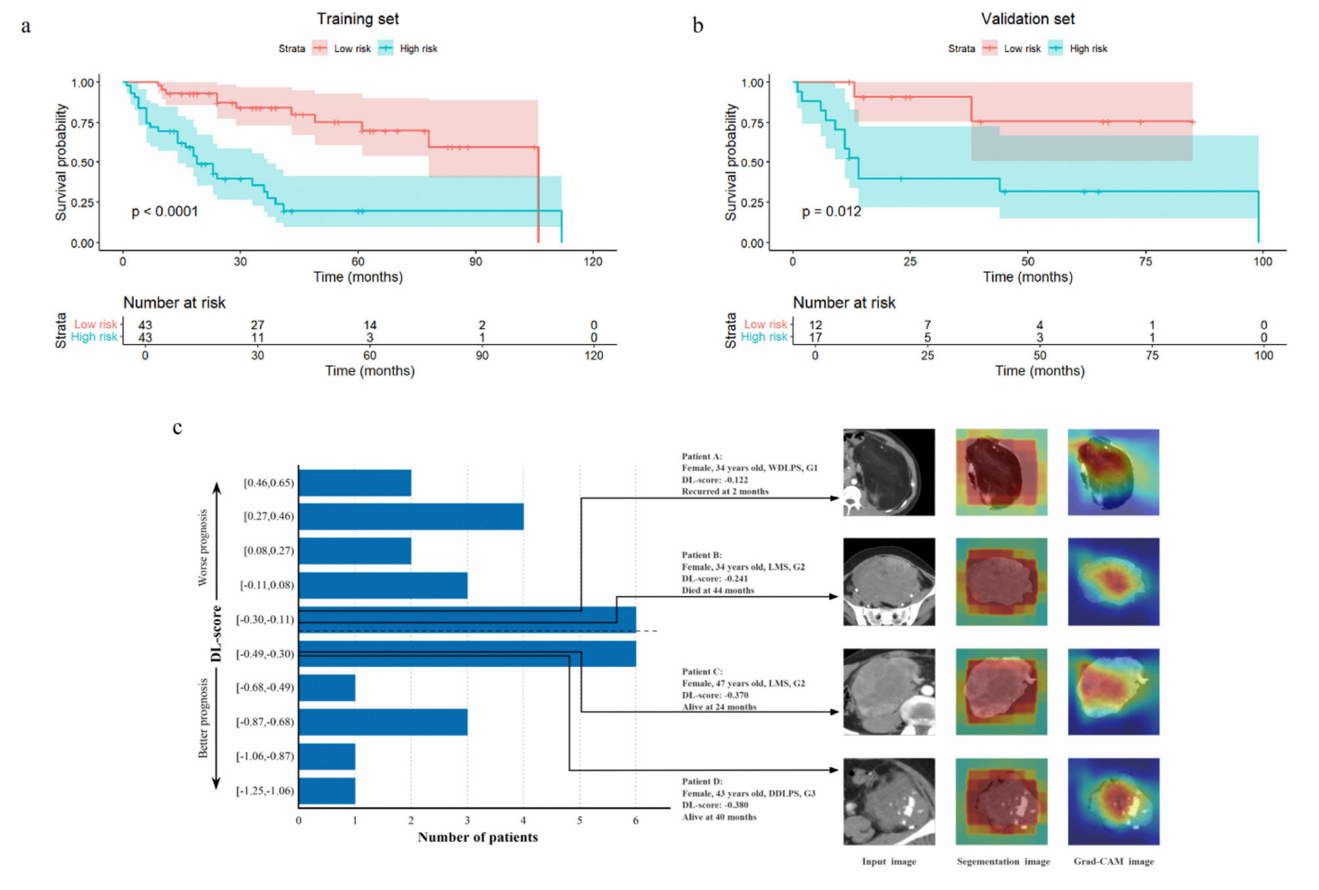
Kaplan-Meier curves of the high-risk group and low-risk group stratified by median DL-score (−0.292) in the training **(a)** and the validation **(b)** sets. The DL-score distribution of the validation set and some examples of original CT images, segementation images and Grad-CAM images **(c)**. The dashed line represents the cutoff value. The highlighted area in the Grad-CAM was mainly focused on the tumor area, indicating high predictive value contributing to model's predictions. DL, deep learning; Grad-CAM, gradient-weighted class activation map; WDLPS, well-differentiated liposarcoma; DDLPS, dedifferentiated liposarcoma; LMS leiomyosarcoma.

This DL-score achieved a C-index of 0.778 (95% CI: 0.708–0.848) with the training set, 0.730 (95% CI: 0.594–0.875) in the validation set, outperforming the clinical model and the Rad-score model (Table 2). It could precisely forecast LRFS at 1, 3, 5 years, with AUC in the validation set of 0.865 (95% CI: 0.727–1.000), 0.855 (95% CI: 0.673–1.000) and 0.749 (95% CI: 0.519–0.979; Figure 2). The DL-score effectively stratified patients into high- and low-risk groups within the training and validation set (log-rank *p* < 0.0001, *p* = 0.012, respectively; Figures 3a, b). Distributions of DL-scores in the validation set and Grad-CAM visualizations of the original images are presented in Figure 3c. Highlighted regions were primarily localized within tumor areas, indicating that the model concentrates on these regions to extract meaningful signatures for LRFS prediction.

### 3.5 Performance of the combined model

**Figure 4:**
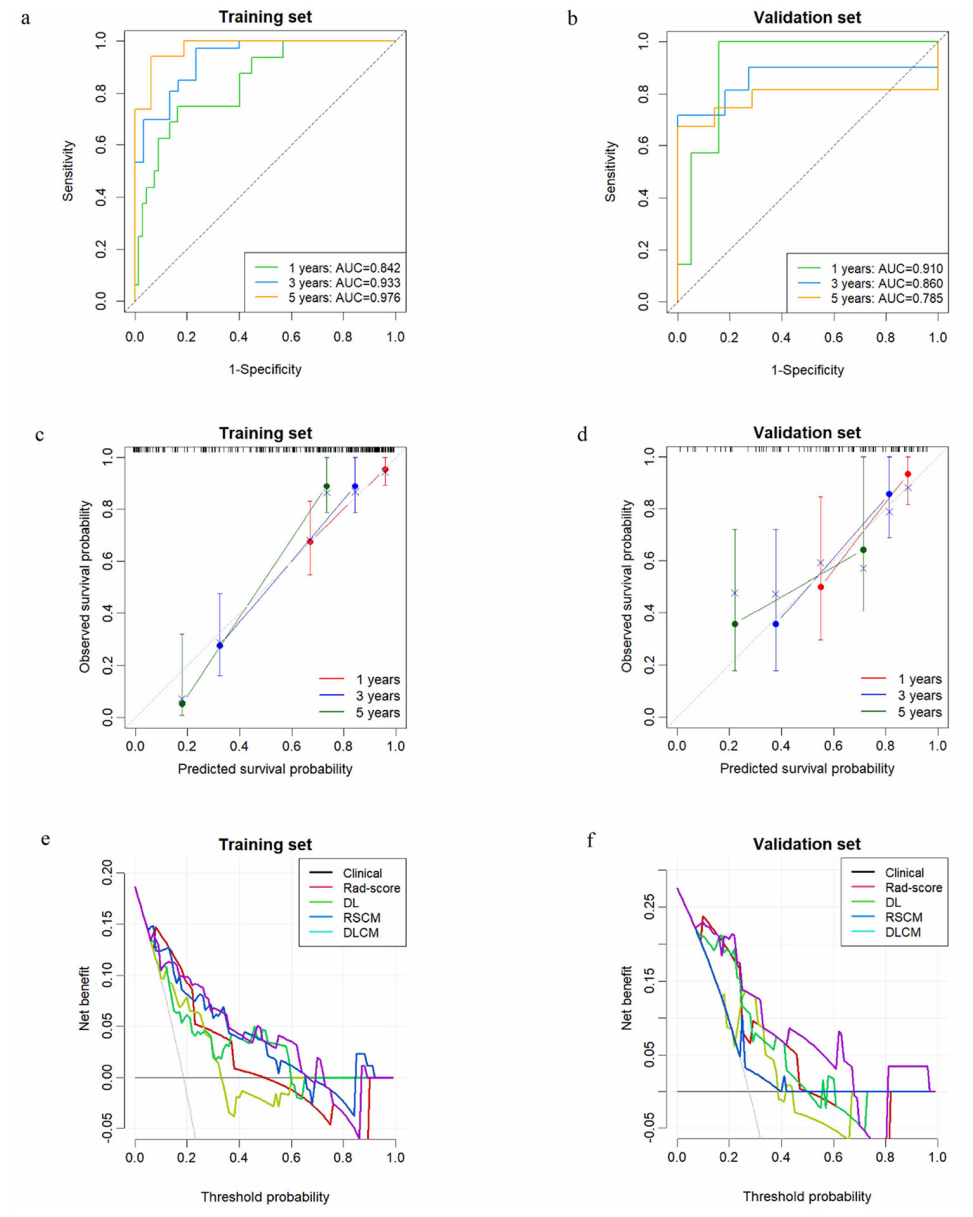
Predictive performance evaluation of the DLCM for LRFS. Time-dependent ROC curves of the DLCM at 1, 3, and 5 years in the training **(a)** and validation **(b)** sets. The calibration plots of the DLCM in the training **(c)** and validation **(d)** sets. The DCA curves of DLCM, RSCM, DL, Rad-score, and clinical models in the training **(e)** and validation **(f)** sets. LRFS, local recurrence-free survival; ROC, receiver operating characteristic; AUC, area under the curve; DCA, decision curve analysis; Rad-score, radiomics score; DL-score, deep learning score; RSCM, the model combined Rad-score with clinical factors; DLCM, the model combined DL-score with clinical factors.

**Figure 5:**
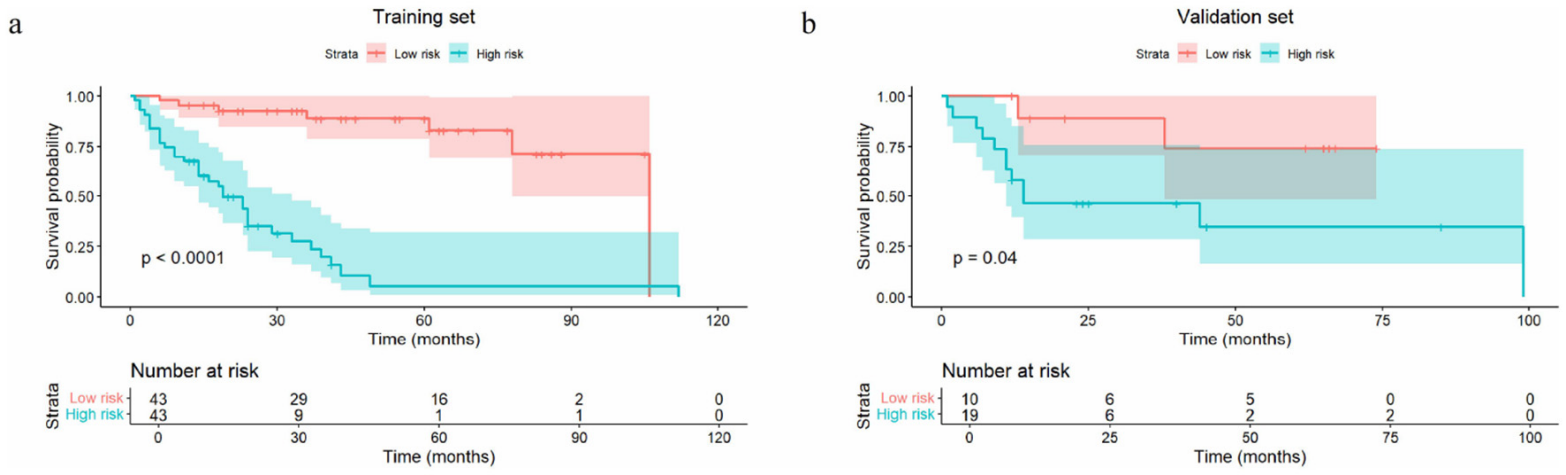
Kaplan-Meier curves of the high-risk group and low-risk group stratified by median DLCM risk score (0.041) in the training **(a)** and the validation **(b)** sets. DLCM, the model combined DL-score with clinical factors.

Multivariate Cox analysis in the training set identified the DL-score as an independent predictor of LRFS (adjusted HR = 5.950, 95% CI: 2.800–12.644; *p* < 0.001; Supplementary Table S2). This RSCM was built by combining the Rad-score with multifocality and FNCLCC grade, while the DLCM was developed by integrating the DL-score, multifocality and FNCLCC grade. The DLCM exhibited superior prediction performance for LRFS compared to each single-modality model and RSCM with the highest C-index of 0.848 (95% CI: 0.790–0.915) within the training dataset and 0.749 (95% CI: 0.601–0.878) within the validation dataset (Table 2). The ROC curves for 1-, 3-, and 5-year LRFS are presented in Figures 4a, b. The calibration plots indicated good consistency between predictions and observed results of the two combined models in the training and validation datasets (Figures 4c, d; Supplementary Figures S4c, d). The DCA further demonstrated that across the relevant threshold range, the DLCM offered a better net benefit compared to the RSCM, DL-score, Rad-score, and clinical models (Figures 4e, f). Using the median risk score (0.041) from the DLCM as the cutoff, patients were divided into high- and low-risk subgroups. As depicted in Figure 5, those in the high-risk category exhibited poorer LRFS within the training (*p* < 0.0001) and validation (*p* = 0.04) sets.

## 4 Discussion

In our research, we constructed an end-to-end DL model that successfully predicted LRFS in RPS patients following curative surgery, using preoperative contrast-enhanced CT scans. This DL model surpassed the conventional handcrafted radiomics and clinical models. The DL-computed risk score (DL-score) served as a significant independent predictor and represents a valuable preoperative risk stratification tool in RPS patients. Furthermore, by integrating the DL-score with clinical predictors—multifocality and FNCLCC grade—the combined DLCM exhibited superior performance over all single-modality models as well as the combined RSCM. Additionally, the risk score calculated from DLCM also proved to be an effective prognostic tool for risk classification. These findings indicate that incorporating imaging-based DL-score can enhance the performance of traditional clinical prognostic models, potentially optimizing treatment and monitoring for RPS patients.

Previous studies on prognosis assessment of RPS have primarily depended on clinical and pathological parameters (2, 5, 7). However, due to the pronounced heterogeneity of RPS, existing models based on these features often demonstrate suboptimal predictive performance. Radiomics can noninvasively mine high-dimensional quantitative features from medical imaging, which can reflect tumor phenotype and providing prognostic information. Arthur et al. (16) successfully constructed a radiomics model to predict RPS histologic types and grades, achieving AUC of 0.928 and 0.882, respectively, within their validation cohort. Pasquali et al. (17) combined manual radiomics features with the Sarculator nomogram to forecast OS and disease-free survival (DFS) among RPS individuals, reaching C-index of 0.726 and 0.639 in the test set. Their results suggest radiomic features only marginally improved accuracy of the Sarculator. In our study, the handcrafted Rad-score model showed moderate performance. This could be attributed to the inherent constraints of handcrafted radiomics features, which rely on pre-defined morphologic and textural features and may fail to capture all critical information—particularly in RPS, where high heterogeneity leads to varied recurrence behavior.

Based on the above considerations, deep learning (DL), an advanced machine learning method (30, 31), enables the direct extraction of more in-depth and comprehensive information from raw images without requiring predefined features. DL has demonstrated superior performance over radiomic analysis in breast cancer (32) and lung cancer (33). Tian et al. (34) constructed a deep learning radiomics nomogram (DLRN) using CT imaging in retroperitoneal leiomyosarcoma (RLS) patients to predict metachronous distant metastasis (MDM), achieving AUC of 0.939 and 0.822 in the training and external validation groups, respectively. Liu et al. (35) developed an MRI-based DLRN to predict tumor relapse in soft tissue sarcomas (STS) patients, reaching C-index between 0.721 and 0.766 in testing. While these studies provided valuable insights, they did not employ fully end-to-end DL frameworks, as they still incorporated manual feature engineering. In contrast, our framework implements a more automated, end-to-end learning process where the model directly maps input pixels to a prognostic score, eliminating intermediary manual feature handling. The superiority over the traditional radiomics model can be explained by the ability of DL to effectively capture the heterogeneity and spatial information in RPS that are often missed by handcrafted radiomics approaches (18). We employed the Cox partial likelihood as the loss function to compute a continuous prognostic risk estimate for each patient, thereby avoiding subjectivity from arbitrary thresholds used to binarize survival time. The model was further regularized through an auxiliary segmentation task, which encouraged the learning of morphologically meaningful features despite its moderate quantitative segmentation performance (25, 26). Grad-CAM visualization enhanced interpretability and transparency by highlighted certain areas of high predictive relevance in the activation maps. These regions likely reflect tumor characteristics such as size, morphology, density, and perfusion, associated with tumor progression.

Some studies (12, 36, 37) have demonstrated that multi-modal strategies can mutually reinforce feature representation and further enhance prediction performance compared to single-modality approaches. In our work, we developed the DLCM by combining clinicopathological factors with the DL-score, which yielded superior efficacy. Although mixing handcrafted features and deep-learning signatures offers potential benefits, it might cause overfitting due to feature redundancy (38). We therefore separately evaluated the added prognostic value of radiomic and DL signatures to the clinical model, further confirming the significant incremental benefit provided by the DL-based signature (39). Utilizing the multimodal DLCM, we succeeded in classifying patients more effectively into distinct high- and low-risk categories. This suggests that for high-risk individuals, follow-up screening should be intensified rather than relying on symptom-triggered examinations, ensuring appropriate interventions can be implemented promptly. Despite a modest C-index reduction in validation, DL-score and DLCM retained C-indices of 0.730 and 0.749—well above the clinical utility threshold and clinically meaningful. Coupled with excellent calibration and clinical net benefit, this confirms their generalizability rather than invalid overfitting.

This research has several limitations. First, its retrospective, single-institution design may lead to selection bias and hidden confounders. Second, the modest sample size may limit model stability and increase overfitting risks, despite the use of data augmentation to improve robustness and generalizability. Third, although Grad-CAM provides a degree of interpretability, the inherent “black-box” characteristic of deep learning models remains a limitation, and its biological meaning requires further clarification. Fourth, manual tumor delineation may be susceptible to interobserver subjective variability, and the reliance on it hinders full automation of the clinical practice workflow. Fifth, the use of 5 representative 2D slices might not fully capture the volumetric tumor heterogeneity, potentially underutilizing 3D spatial information—a compromise necessitated by the limited availability of well-validated pre-trained 3D models in medical imaging. Therefore, future work will focus on the construction of 3D models and external validation in prospective multicenter studies to enhance the clinical utility of our framework.

## 5 Conclusion

In summary, we constructed and evaluated an end-to-end DL model based on preoperative CT, which successfully predicts LRFS in RPS patients following curative resection. Furthermore, the combined DLCM model, which integrates DL-score, demonstrated superior predictive performance and provided considerable prognostic stratification value. Our model offers a noninvasive tool to preoperatively assess LRFS in RPS patients, potentially guiding personalized treatment plans and follow-up strategies.

## Data Availability

The original contributions presented in the study are included in the article/Supplementary material, further inquiries can be directed to the corresponding authors.
