# Supplementary material for: A CT-based deep learning model to predict local recurrence-free survival in primary retroperitoneal sarcoma
Source: Front Med (Lausanne). 2026 Jan 2;12:1725377. doi: 10.3389/fmed.2025.1725377 (PMC12808379; doi:10.3389/fmed.2025.1725377)
Supplement: Supplementary file 1 [file Data_Sheet_1.docx]

**List of Appendix**

**Part I. Supplementary Contents**

**Supplemental Content 1:** The inclusion and exclusion criteria of patients

**Supplemental Content 2:** CT scanning parameters

**Supplemental Content 3:** Hand-crafted radiomics feature extraction

**Supplemental Content 4:** Hand-crafted radiomics feature selection and model construction

**Supplemental Content 5:** Introduction for CBAM

**Supplemental Content 6:** Details of Cox loss

**Part II. Supplementary Figures**

**Fig. S1:** Predictive efficacy of clinical model on LRFS

**Fig. S2:** Feature selection for the Rad-score model using LASSO-Cox regression

**Fig. S3:** Predictive efficacy of Rad-score model on LRFS

**Fig. S4:** Predictive performance evaluation of the RSCM for LRFS

**Part III. Supplementary Tables**

**Table S1:** Univariate and multivariate Cox analysis of clinical factors for LRFS in the training set

**Table S2:** Multivariate Cox analysis of DL-score, Rad-score and other clinical factors for LRFS in the training set

**Table S3:** Comparisons in NRI and IDI indices across different models within the training and validation sets

**Table S4:** The Dice scores of deep learning model's auxiliary segmentation head

**Part I. Supplemental Contents**

**Supplemental Content 1: The inclusion and exclusion criteria of patients**

Inclusion criteria were as follows: (1) pathologically confirmed primary RPS (including well-differentiated liposarcoma [WDLPS], dedifferentiated liposarcoma [DDLPS], leiomyosarcoma [LMS], and other subtypes such as solitary fibrous tumor [SFT], malignant peripheral nerve sheath tumor [MPNST], synovial sarcoma (SS), or undifferentiated pleomorphic sarcoma [UPS]); (2) underwent radical surgical resection; (3) contrast-enhanced CT available within 2 weeks before surgery. Exclusion criteria were: (1) receipt of neoadjuvant therapy before baseline CT scans; (2) other pathological types (e.g., extraosseous Ewing sarcoma, desmoplastic small round cell tumor, ligamentous fibroma, uterine sarcoma, gastrointestinal stromal tumors); (3) with other concurrent malignant neoplasms; (4) lack of CT data or poor image quality; (5) incomplete clinical and follow-up data.

**Supplemental Content 2: CT scanning parameters**
Contrast-enhanced CT scans were conducted using the following helical CT scanners: Philips Healthcare or GE Medical Systems.The following scanning parameters were employed: tube voltage, 120 kV; tube current, 240–340 mA; slice thickness, 1-5 mm. Contrast medium (iohexol) was intravenously injected at a dose of 1.5 mL/kg body weight and a rate of 3 ml/s. Arterial phase images were collected after contrast material injection with a delay of 25-30s in each patient.

**Supplemental Content 3: Hand-crafted radiomics feature extraction**

For the construction of the hand-crafted radiomics model, features were extracted from the regions of interest (ROIs) of arterial phase contrast-enhanced CT images using the open-source Python package Pyradiomics. The extracted features encompass the following categories: (1) shape features, comprising three-dimensional descriptors that reflect the shape and size of the ROIs; (2) first-order features, representing commonly used metrics that quantitatively describe the distribution of voxel intensities within ROIs; (3) texture features, also known as “second-order” features, calculated from gray level co-occurrence matrix (GLCM), gray level run length matrix (GLRLM), gray level size zone matrix (GLSZM), gray level dependence matrix (GLDM), and neighboring gray tone difference matrix (NGTDM), which describe spatial complexity and intensity relationships among adjacent voxels; and (4) filter- and wavelet-based features, encompassing both intensity and texture features derived from filtered and transformed versions of the original images, including exponential, logarithm, square, square root, gradient, local binary pattern (lbp_2D, lbp_3D_k), and various wavelet decompositions (wavelet-LHL, wavelet-LHH, wavelet-HLL, wavelet-LLH, wavelet-HLH, wavelet-HHH, wavelet-HHL, and wavelet-LLL).

**Supplemental Content 4: Hand-crafted radiomics feature selection and model construction**

(1) Features demonstrating high stablity (ICCs > 0.9) were retained for subsequent analysis. (2) All radiomics features were standardized using z-score normalization in the training cohort, and the same scaling parameters were applied to the validation set. Features with near-zero variance or constant values were excluded. (3) Features significantly associated with local recurrence-free survival (LRFS) were indentified using univariate Cox proportional hazards regression (p < 0.05). (4) Highly redundant features (Spearman's rank correlation coefficient > 0.80) were excluded to mitigate multicollinearity. (5) Subsequently, least absolute shrinkage and selection operator (LASSO) Cox regression with 10-fold cross-validation (lambda.min) was used to select candidate features with non-zero coefficients. (6) These selected features then underwent bidirectional stepwise multivariate Cox regression based on the Akaike Information Criterion (AIC), with variables capped at ≤ 1/10 of events to prevent overfitting. The final Rad-score was computed as a linear combination of selected features weighted by their multivariate Cox coefficients.

**Supplemental Content 5:** **Introduction for CBAM**

Convolutional Block Attention Module (CBAM), proposed by Sanghyun Woo et al. ^[1]^ in 2018, is an attention mechanism module to improve the perceptual ability of convolutional neural networks (CNNs). CBAM consists of two main attention modules: the channel attention module (CAM), which enhances the feature representation of different channels, and the spatial attention module (SAM), which aids in extracting critical information from different spatial locations. CBAM can be ultilized as a layer and inserted at any convolutional block of a CNN architecture.

**Supplemental Content 6: Details of Cox loss**

The Cox loss is derived from the partial likelihood of the Cox proportional hazards model, a semi-parametric method widely adopted in survival analysis. The partial likelihood centers on capturing the probability of the observed order of events and the corresponding risk scores for each event. By maximizing this likelihood function, the model estimates the coefficients that quantify the influence of covariates on the hazard function. The loss function is formulated as follows:

$$\text{L}\left( \text{θ} \right)\text{=−}\sum_{\text{i:Ei=1}} \left( {\hat{\text{h}}}_{\text{θ}}\left( \text{x}_{\text{i}} \right)\text{−log}\sum_{\text{j}\text{∈ℜ}\left( \text{T}_{\text{i}} \right)} \text{e}^{{\hat{\text{h}}}_{\text{θ}}\left( \text{x}_{\text{j}} \right)}\text{ } \right)$$

where the i and j denote indices of individual samples; $\text{Ei}$=1 indicates the occurrence of the terminal event; x refers the input data; $\hat{h}_{\theta}\left( x \right)$ denotes to the continuous risk score output by the model; $\text{ℜ}\left( \text{T}_{\text{i}} \right)$ denotes the set of all samples at risk at the time when the event occurs.

**Appendix Reference**

[1] Woo S, Park J, Lee JY, Kweon IS. CBAM: Convolutional Block Attention Module. Lecture Notes in Computer Science. 2018 : 3-19.

**Part II. Supplemental Figures**





**Fig. S1** Predictive efficacy of clinical model on LRFS. Time-dependent ROC curves of the clinical model in the training (a) and the validation (b) sets. LRFS, local recurrence-free survival; ROC, receiver operating characteristic; AUC, area under the curve.





**Fig. S2** Feature selection for the Rad-score model using LASSO-Cox regression. (a) 10-fold cross-validation curve. The red vertical dashed line marks λₘᵢₙ that minimizes the cross-validation error, which was used to further select features with non-zero coefficients. (b) Coefficient path plot. The red vertical dashed line corresponds to the λₘᵢₙ from (a), where features with non-zero coefficients represent the candidate features selected by LASSO. Rad-score, radiomics score; LASSO, least absolute shrinkage and selection operator.





**Fig. S3** Predictive efficacy of Rad-score model on LRFS. Time-dependent ROC curves of the Rad-score model in the training (a) and the validation (b) sets. Rad-score, radiomics score; LRFS, local recurrence-free survival; ROC, receiver operating characteristic; AUC, area under the curve.





**Fig. S4** Predictive performance evaluation of the RSCM for LRFS. Time-dependent ROC curves of the RSCM at 1, 3, and 5 years in the training (a) and validation (b) sets. The calibration plots of the RSCM in the training (c) and validation (d) sets. LRFS, local recurrence-free survival; ROC, receiver operating characteristic; AUC, area under the curve; RSCM, the model combined Rad-score with clinical factors.

**Part III. Supplemental Tables**

**Table S1** Univariate and multivariate Cox analysis of clinical factors for LRFS in the training set

| **Variables** | **Univariate** | |  | **Multivariate** | |
| --- | --- | --- | --- | --- | --- |
|  | **HR (95% CI)** | **p value** |  | **HR (95% CI)** | **p value** |
| Sex | 0.886 (0.469-1.673) | 0.709 |  |  |  |
| Age | 0.984 (0.960-1.008) | 0.189 |  |  |  |
| BMI | 0.977 (0.883-1.082) | 0.659 |  |  |  |
| Tumor size (cm) | 1.022 (0.993-1.052) | 0.141 |  |  |  |
| Multifocality | 2.440 (1.149-5.182) | **0.020** |  | 2.795 (1.277-6.114) | **0.010** |
| Histology subtypes |  |  |  |  |  |
| WDLPS |  |  |  |  |  |
| DDLPS | 4.216 (1.751-10.151) | **0.001** |  |  |  |
| LMS | 1.428 (0.533-3.826) | 0.478 |  |  |  |
| Others | 1.162 (0.528-2.554) | 0.709 |  |  |  |
| FNCLCC grade |  |  |  |  |  |
| G1 |  |  |  |  |  |
| G2 | 3.136 (1.645-5.982) | **0.001** |  | 2.995 (1.568-5.722) | **<0.001** |
| G3 | 0.830 (0.478-1.440) | 0.507 |  | 0.724 (0.409-1.280) | 0.266 |
| Adjuvant treatment | 1.511 (0.774-2.949) | 0.226 |  |  |  |

LRFS, local recurrence-free survival; HR, hazard ratio; CI, confidence interval; WDLPS, well-differentiated liposarcoma; DDLPS, dedifferentiated liposarcoma; LMS leiomyosarcoma; FNCLCC, Federation Nationale des Centres de Lutte Contre le Cancer.

**Table S2** Multivariate Cox analysis of DL-score, Rad-score and other clinical factors for LRFS in the training set

| **Variables** | **Multivariate analysis** | | |
| --- | --- | --- | --- |
|  | **HR** | **95% CI** | **p value** |
| Sex | 0.778 | (0.348-1.737) | 0.540 |
| Age | 0.999 | (0.967-1.032) | 0.954 |
| BMI | 0.905 | (0.790-1.036) | 0.148 |
| Tumor size (cm) | 1.009 | (0.964-1.055) | 0.714 |
| Multifocality | 3.328 | (1.378-8.034) | **0.008** |
| Histology subtypes |  |  |  |
| WDLPS |  |  |  |
| DDLPS | 2.700 | (0.689-10.586) | 0.154 |
| LMS | 0.939 | (0.212-4.158) | 0.934 |
| Others | 0.790 | (0.273-2.287) | 0.664 |
| FNCLCC grade |  |  |  |
| G1 |  |  |  |
| G2 | 2.670 | (1.165-6.122) | **0.020** |
| G3 | 0.745 | (0.319-1.740) | 0.496 |
| Adjuvant treatment | 1.269 | (0.557-2.889) | 0.571 |
| Rad-score | 1.327 | (0.627-2.810) | 0.460 |
| DL-score | 5.950 | (2.800-12.644) | **<0.001** |

LRFS, local recurrence-free survival; HR, hazard ratio; CI, confidence interval; WDLPS, well-differentiated liposarcoma; DDLPS, dedifferentiated liposarcoma; LMS leiomyosarcoma; FNCLCC, Federation Nationale des Centres de Lutte Contre le Cancer; Rad-score, radiomics score; DL-score, deep learning score.

**Table S3** Comparisons in NRI and IDI indices across different models within the training and validation sets

| **Models** | **Training set** | | | | |  | **Validation set** | | | |
| --- | --- | --- | --- | --- | --- | --- | --- | --- | --- | --- |
|  | **NRI**  **(95% CI)** | **p value** |  | **IDI**  **(95% CI)** | **p value** |  | **NRI**  **(95% CI)** | **p value** | **IDI**  **(95% CI)** | **p value** |
| Clinical | Ref |  |  | Ref |  |  | Ref |  | Ref |  |
| Rad-score | 0.429 (-0.076-0.927) | 0.12 |  | 0.007 (-0.102-0.125) | 0.820 |  | 0.152 (-0.359-0.800) | 0.848 | 0.079 (-0.219-0.412) | 0.748 |
| DL-score | 0.997 (0.431-1.444) | **<0.001** |  | 0.152 (0.007-0.278) | **0.044** |  | 1.162 (0.295-1.871) | **0.012** | 0.146 (0.026-0.261) | **0.016** |
| RSCM | 0.839 (0.346-1.335) | **<0.001** |  | 0.102 (0.040-0.167) | **<0.001** |  | 0.152  (-0.370-0.852) | 0.892 | 0.078  (-0.221-0.471) | 0.768 |
| DLCM | 1.226 (0.742-1.605) | **<0.001** |  | 0.285 (0.182-0.388) | **<0.001** |  | 1.143 (0.400-2.000) | **0.036** | 0.141 (0.057-0.228) | **<0.001** |

NRI, net reclassification improvement; IDI, integrated discrimination improvement; Rad-score, radiomics score; DL-score, deep learning score; RSCM, the model combined Rad-score with clinical factors; DLCM, the model combined DL-score with clinical factors.

**Table S4** The Dice scores of deep learning model's auxiliary segmentation head

|  | **Dice Mean** | **Dice Std** | **Dice Min** | **Dice Max** |
| --- | --- | --- | --- | --- |
| Training set | 0.575 | 0.018 | 0.548 | 0.601 |
| Validation set | 0.633 | 0.008 | 0.625 | 0.641 |
